# A Dietitian-Led Vegan Program May Improve GlycA, and Other Novel and Traditional Cardiometabolic Risk Factors in Patients With Dyslipidemia: A Pilot Study

**DOI:** 10.3389/fnut.2022.807810

**Published:** 2022-03-24

**Authors:** Tina H. T. Chiu, Yun-Chun Kao, Ling-Yi Wang, Huai-Ren Chang, Chin-Lon Lin

**Affiliations:** ^1^Department of Nutritional Science, Fu-Jen Catholic University, New Taipei City, Taiwan; ^2^Department of Nutrition Therapy, Dalin Tzu Chi Hospital, Buddhist Tzu Chi Medical Foundation, Chiayi, Taiwan; ^3^Epidemiology and Biostatistics Consulting Center, Department of Medical Research, Hualien Tzu Chi Hospital, Buddhist Tzu Chi Medical Foundation, Hualien, Taiwan; ^4^Department of Pharmacology, School of Medicine, Tzu Chi University, Hualien, Taiwan; ^5^Department of Pharmacy, Hualien Tzu Chi Hospital, Buddhist Tzu Chi Medical Foundation, Hualien, Taiwan; ^6^Division of Cardiology, Department of Internal Medicine, Hualien Tzu Chi Hospital, Buddhist Tzu Chi Medical Foundation, Hualien, Taiwan; ^7^School of Medicine, Tzu Chi University, Hualien, Taiwan; ^8^Division of Cardiology, Department of Internal Medicine, Dalin Tzu Chi Hospital, Buddhist Tzu Chi Medical Foundation, Chiayi, Taiwan

**Keywords:** GlycA, lipoprotein particles, TMAO, vegan, cardiometabolic risk factors

## Abstract

**Background:**

Systematic inflammation and lipid profiles are two major therapeutic targets for cardiovascular diseases. The effect of a nutritionally balanced vegan diet on systematic inflammation and lipoprotein subclass awaits further examination.

**Objective:**

To investigate the change in novel and traditional cardiometabolic risk factors before and after a dietitian-led vegan program, and to test the bioavailability of vitamin B12 in Taiwanese purple laver as part of a vegan diet.

**Design:**

A one-arm pilot intervention study.

**Participants/Setting:**

Nine patients with dyslipidemia participated in this 12-week vegan program.

**Main Outcome Measures:**

Nuclear Magnetic Resonance (NMR) detected GlycA signals (systematic inflammation) and lipoprotein subclass (atherogenicity); trimethylamine N-oxide (TMAO); and other cardiometabolic risk factors.

**Statistical Analyses Performed:**

Wilcoxon signed-rank test.

**Results:**

In this 12-week vegan intervention emphasizing whole foods, systematic inflammation improved as indicated by a reduction in GlycA (median: −23 μmol/L, *p* = 0.01). LDL-c (low-density lipoprotein cholesterol) (median −24 mg/dl, *p* = 0.04) and LDL-p (low-density lipoprotein particles) (median −75 nmol/L, *p* = 0.02) both decreased significantly. VLDL (very-low-density lipoprotein) and chylomicron particles showed a decreasing trend (−23.6 nmol/L, *p* = 0.05). Without caloric restriction, body mass index (BMI) (−0.7 kg/m^2^, *p* = 0.03), waist circumferences (−2.0 cm, *p* < 0.001), HbA1c (−0.2%, *p* = 0.02), and (HOMA-IR) homeostatic model assessment for insulin resistance (−0.7, *p* = 0.04) have all improved. The change in the TMAO and vitamin B12 status as measured by holo-transcobalamin appeared to depend on baseline diets, TMAO, and vitamin B12 status.

**Conclusions:**

A dietitian-led vegan program may improve systematic inflammation and other novel and traditional cardiometabolic risk factors in high-risk individuals.

## Introduction

Cholesterol and inflammation are two of the most important risk factors and major treatment targets for coronary heart diseases. Although statin is a powerful drug to lower cholesterol and inflammation (1), it may increase diabetes risk in high-risk individuals (2), potentially related to altered lipoprotein subclass distribution (3). Anti-inflammatory therapy using Canakinumab could post serious side effects such as neutropenia and infection-related death (4). Dietary interventions that simultaneously reduce inflammation and atherogenic lipids, and improve glucose metabolism may be a holistic primary treatment or an adjuvant to pharmaceutical therapies.

Vegetarian and vegan diets are associated with better lipid profiles and a lower risk of ischemic heart diseases and diabetes (5). A recent Cochrane analysis found that vegan diets reduce the total cholesterol (TC) and low-density lipoprotein cholesterol (LDL-c), though raise triglyceride (TG) and lower high-density lipoprotein cholesterol (HDL-c) slightly (6). Emerging evidence suggests that lipoprotein particles may be more predictive of cardiovascular outcomes than lipoprotein concentrations, and low-density lipoprotein particles (LDL-p) better account for residual risk after statin treatment than LDL-c (7, 8). Detailed examination of subtypes of lipoproteins, such as very-low-density lipoprotein (VLDL) and chylomicron particles, and different sizes of LDL-p and high-density lipoprotein particles (HDL-p), may also yield insight on the risk of diabetes (3, 9). The effect of vegan diets on lipoprotein particles has never been examined.

Vegetarian and vegan diets have generally been found to be anti-inflammatory (10) but could be proinflammatory when the vitamin B12 status is compromised (11). GlycA, measured by NMR signals, is a novel composite biomarker for inflammation, that quantifies both protein concentrations and the glycosylation state of several acute-phase proteins, is predictive of future cardiovascular disease outcomes, and has low intra-personal variability (12, 13). GlycA has been associated with impaired insulin secretion and is a better predictor for incident diabetes and cardiovascular events than CRP (C-reactive protein) and interleukin-1 receptor antagonist (14). While several nutrients have been correlated with GlycA (15, 16), the impacts of overall dietary patterns on GlycA have yet to be examined.

Vegetarian diets may also influence cardiometabolic health through their interplay with the gut microbiota through the manifestation of trimethylamine N-Oxide (TMAO) (17). TMAO could inhibit reverse cholesterol transport (18), induce platelet hyperreactivity and thrombosis (19), and have been associated with cardiovascular events and all-cause mortality in a dose-response manner (20). Thus, examining changes in TMAO may add extra insight into the cardiometabolic effects of a vegan diet.

Most epidemiological studies define vegetarian or vegan diets by the avoidance of animal-based foods rather than the composition and quality of the diets. Although the association between coronary heart diseases and the quality of plant-based diets (healthy, unhealthy, or overall plant-based diets) have been studied (21), such dietary scoring system is based on the consumption frequency of food groups and does not address nutrients of concerns for vegetarians (such as vitamin B12, omega-3 fatty acid, and other nutrients) (22). The anti-atherogenic potential of a healthy vegan diet planned by registered dietitians to support nutritional adequacy has yet to be examined.

One of the nutrients of concern for vegans is vitamin B12. Sea vegetables have been regarded as an unreliable source of vitamin B12 (22), though Korean/Japanese nori and Taiwanese purple laver have been suggested to contain true vitamin B12 rather than pseudo vitamin B12 (23). The bioavailability of these sea vegetables needs to be tested in human interventions.

This study aimed to examine the effect of a healthy vegan diet (using Taiwanese purple laver as a source of vitamin B12), on several novel markers of cardiovascular diseases, including GlycA, lipoprotein particles and size, TMAO, and other traditional cardiometabolic risk factors.

## Methods

### Study Design and Participants

**Figure 1:**
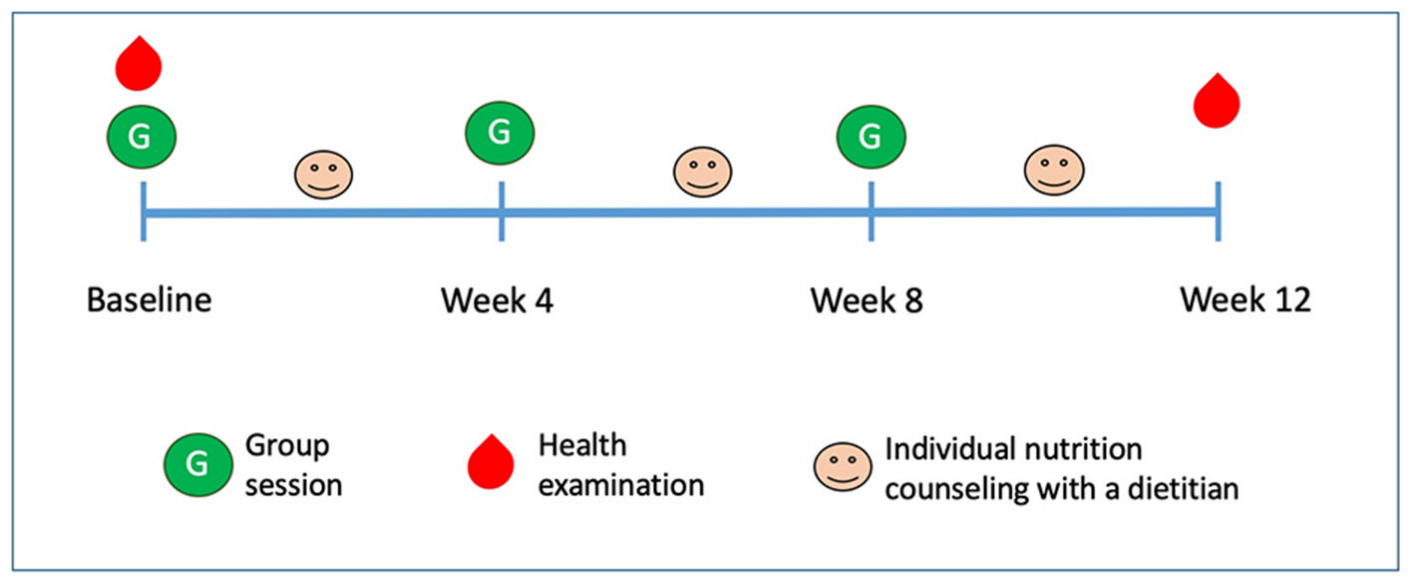
Study design and timeline overview. Participants received three group sessions and three individual nutrition counseling sessions with a dietitian. Health examination (including anthropometrics and blood examination) were performed at baseline and end of week 12.

This one-arm intervention study lasted for 12 weeks (from March 28 to June 14, 2018). Figure 1 depicts the study design: health examinations were performed at baseline and the end of week 12; participants joined three monthly group lessons and three individual nutrition counseling sessions with a registered dietitian.

The recruitment criteria includes adults aged 20 to 75 years old, with dyslipidemia (at least one of the following: TG ≥ 150 mg/dl, TC ≥ 200 mg/dl, LDL-c≥ 130 mg/dl, HDL-c < 45 mg/dl for male or < 55 mg/dl for female, or receiving statin treatment). Participants were excluded if they had chronic kidney disease, history of coronary artery disease or ischemic heart disease, heart failure, stroke, or cancer of any type. A total of ten patients meeting the above criteria and willing to participate in the study were recruited from the cardiology clinic at the Hualien Tzu Chi Hospital, Hualien, Taiwan, by coauthor Chang. One dropped out during the study (did not return for final health examination). The study was approved by the Institutional Review Board of Hualien Tzu Chi General Hospital.

### The Study Diet

The dietary intervention emphasized brown rice and whole grains, vegetables (400–600 g daily), fruits (at least three servings daily), beans and soy (at least seven servings daily), and nuts (at least three servings daily); while avoiding all animal products (meat, fish and seafood, eggs, dairy), added sugar, fried foods, refined carbohydrates (white rice, white bread, and pastry), ultra-processed foods, and with no restriction on energy intakes. In addition, participants were provided with and instructed to eat these foods: flaxseed meal, walnuts, pecans, extra-virgin olive oil, Brazil nuts, and Taiwanese purple laver (also known as “hong-mao-tai” or *Bangia atropurpurea*, and was selected to test for its vitamin B12 bioavailability). These foods were provided by the study to address nutrients of concern relevant to a vegan diet. The prescribed amount and nutrient composition of these foods, calculated based on the Taiwan Food Composition Database (24) and the United States Department of Agriculture Food Composition database (25), could be found in Table 1.

**Table 1:** Amount and nutrient contents of foods provided in the intervention.

**Foods**	**Portion**	**Energy (kcal)**	**Iron (mg)**	**Zinc (mg)**	**Vitamin B12 (μg)**	**Alpha linolenic acid (mg)**	**Selenium (μg)**
Flaxseed	15g/day	84	1.0	0.8	0	3791	
Walnuts	10.5g/day	70	0.3	0.3	0	747	
Pecans^*^	7g/day	48	0.2	0.3	0	69	
Taiwanese purple laver	5g/day	13	3.1	0.2	3.7	6	
Olive oil	15g/d	132	0.0	0.0	0	99	
Brown rice (raw)	at least 80g/d	287	0.8	1.8	0	33	
Brazil nuts^*^	1 nut (7g)/week	46	0.1	0.3	0	3	134
	Sum	680	5.5	3.7	3.7	4748	134.0

* *Values based on United States Department of Agriculture Food Composition Database (25); all other values are based on Taiwan Food Composition Table (24)*.

### Health Examinations

Health examinations were performed at baseline and at the end of week 12. Anthropometric measurements including height, weight, and waist circumferences were taken by a trained researcher. Venous blood was drawn after an overnight fast and sent immediately to the Department of Laboratory Medicine at the Hualien Tzu Chi Hospital (Hualien, Taiwan) to assess routine clinical lipid profiles and glucose (Dimension RxL, Siemens Healthcare GmbH, Erlangen, Germany), insulin (ADVIA Centaur XP, Siemens Healthcare GmbH, Erlangen, Germany), glycated hemoglobin (HbA1C) (BIO-RAD VARIANT II TURBO, Bio-Rad Laboratories, Inc, Hercules CA, USA), and hemoglobin (XE-5000, Sysmex Europe GmbH, Norderstedt, Germany). The remaining blood samples were centrifuged and stored at a −80°C freezer.

Nuclear Magnetic Resonance (NMR)-based GlycA, and lipoprotein particles and particle sizes were analyzed by LabCorp (Morrisville, NC, USA). Plasma TMAO, choline, and carnitine were analyzed using Agilent 1,290 UHPLC (ultra high performance liquid chromatography) coupled with an Agilent 6,460 triple quadrupole mass spectrometer (Agilent Technologies, Santa Clara, CA, USA) by the National Taiwan University Center of Genomics and Precision Medicine (Taipei, Taiwan). The plasma holotranscobalamin was analyzed using an ELISA kit (IBL International GmbH, Germany, kit serial number: 902931842) and the oxidized LDL-cholesterol was also analyzed using an ELISA kit (Mercodia, Sweden, kit serial number: 31,139) both by Yi-Her Laboratory (Yilan, Taiwan). Homeostatic model assessment (HOMA) was used to estimate insulin resistance (HOMA-IR) {[Glucose (mg/dl) × insulin (mIU/L)]/405} and beta-cell function {HOMA-beta: ([20 × insulin (mIU/L)]/[Glucose (mg/dl)-63])} using fasting glucose and insulin (26).

### Collection of Other Variables

Medical history and uses of medication were obtained from medical charts by coauthor Chang. Physical activities and sleep time were assessed using the International Physical Activity Questionnaire (IPAQ) Taiwan Show-Card version (27).

### Statistical Analysis

Wilcoxon signed-rank test was used to assess the changes in cardiometabolic risk factors before and after dietary intervention. The SAS Statistical Software (version 9.4, SAS Institute, Cary, NC, USA) was used to perform the statistical analysis.

## Results

Table 2 summarizes the baseline characteristics of the participants (*n* = 9) who completed the pilot study. The age of the participants was averaged to 58 (range: 44–77) years. All participants were diagnosed with dyslipidemia (56% on a statin), six with hypertension (all on antihypertensive medications), and two with diabetes (both on oral glucose-lowering agents). Medications did not change during the intervention except for one patient who was taken off anti-hypertensive medication due to a substantial decrease in blood pressure during the diet intervention. Three participants were Lacto-ovo vegetarians prior to joining the study.

**Table 2:** Baseline characteristics of nine study participants.

	**Mean (SD) or n**
Age, years	58.4 (10.0)
Sex	
Male	2
Female	7
Weight status	
Normal (BMI<24.0)	2
Overweight (BMI 24.0–26.9)	1
Obese (BMI>27.0)	6
Abdominal obesity	7
Physical activities, METs/d	471 (539)
Sleep time, hours/day	6.4 (0.5)
Dietary pattern	
Nonvegetarian	6
Vegetarian	3
Medical history	
Diabetes	2
Hypertension	6
Dyslipidemia	9
Medication use	
Glucose lowering agents	2
ACEI/ARB	6
Statin	5
Aspirin	2

*SD, standard deviation; BMI, body mass index; Mets, metabolic equivalents; ACEI, angiotensin converting enzyme inhibitor; ARBs, angiotensin receptor blockers*.

**Figure 2:**
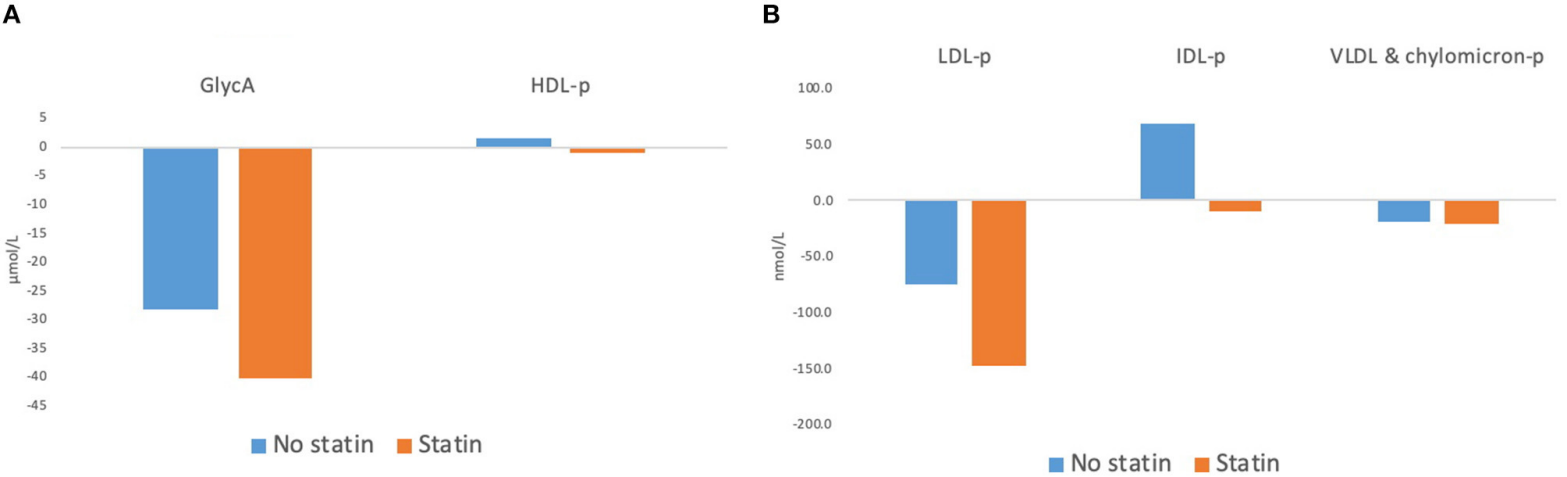
Changes in **(A)** GlycA and HDL-particles and **(B)** LDL-particles, IDL-particles, and VLDL and chylomicron particles after 12 weeks of dietary intervention, with stratification by use of Statin Therapy. HDL-p, high density lipoprotein particles; LDL-p, low-density lipoprotein particles; IDL, intermediate-density lipoprotein particles; VLDL & chylomicron-p, very-low-density lipoprotein and chylomicron particles.

Table 3 shows the NMR-spectroscopy analysis at baseline and after the 12-week intervention. GlycA decreased in eight out of nine participants, and by an overall median of 23 μmol/L or 9% (*p* = 0.01). LDL-p decreased by 75 nmol/L or 8% (*p* = 0.02) mainly accounted for by changes in large LDL-p. VLDL and chylomicron particles (−23.6 nmol/L or 28%, *p* = 0.05) and their TG content (−18 mg/dl, *p* = 0.09) both showed decreasing trends. Although HDL-p did not change significantly, the total, large, and medium HDL-p showed an increasing trend, and the small HDL-p decreased. Figures 2A,B show the changes in GlycA and lipoprotein particles stratified by statin therapy. The sample size was too small to perform statistical analysis or test for interaction.

**Table 3:** Nuclear magnetic resonance spectral analysis of GlycA and lipoprotein particles and size of the nine participants.

	**Baseline**			**12 weeks**			**Changes (12 weeks - baseline)**			
	**Median**	**P25**	**P75**	**Median**	**P25**	**P75**	**Median**	**P25**	**P75**	***p*** **-value**
GlycA, μmol/L	378.0	340.0	420.0	348.0	317.0	374.0	−23.0	−64.0	−17.0	0.01
VLDL and chylomicron										
Total, nmol/L	84.1	81.6	93.1	70.9	49.9	76.4	−23.6	−34.6	0.2	0.05
Large, nmol/L	4.0	3.3	8.0	3.0	2.4	5.1	−0.4	−3.3	0.1	0.20
Medium, nmol/L	33.5	21.2	47.1	21.8	15.1	28.1	−4.6	−19.6	−1.8	0.10
Small, nmol/L	44.2	35.6	53.0	35.9	30.8	38.1	−15.8	−25.0	5.2	0.25
IDL particles, nmol/L	35.0	29.0	123.0	72.0	60.0	142.0	25.0	10.0	57.0	0.20
LDL particles										
Total, nmol/L	1000.0	835.0	1337.0	822.0	799.0	1150.0	−75.0	−187.0	−34.0	0.02
Large, nmol/L	322.0	163.0	508.0	100.0	17.0	239.0	−153.0	−222.0	−131.0	0.01
Small, nmol/L	765.0	509.0	849.0	709.0	641.0	812.0	14.0	−87.0	47.0	0.89
HDL particles										
Total, μmol/L	33.7	30.6	35.3	34.0	30.8	34.8	0.5	−0.5	2.3	0.74
Large, μmol/L	7.5	5.0	8.7	6.9	5.9	7.8	0.3	−1.3	1.0	0.98
Medium, μmol/L	6.3	4.0	8.1	6.4	5.6	8.0	1.1	−0.2	4.4	0.38
Small, μmol/L	22.1	15.8	23.5	19.4	14.2	22.5	−1.1	−4.3	1.6	0.50
Size										
VLDL, nm	47.5	45.7	50.8	47.6	44.9	48.7	−0.8	−4.5	3.1	0.57
LDL, nm	20.2	20.2	20.7	20.0	19.8	20.2	−0.3	−0.4	−0.2	0.94
HDL, nm	9.3	8.8	9.9	9.3	9.3	9.5	0.0	−0.3	0.2	0.02
Triglyceride, mg/dL^*^	159.0	129.0	188.0	120.0	77.0	168.0	−20.0	−86.0	−3.0	0.10
VDLD and chylomicron TG, mg/dL^*^	112.0	98.0	133.0	93.0	70.0	115.0	−18.0	−60.0	−2.0	0.09
HDL cholesterol, mg/dL^*^	49.0	47.0	52.0	47.0	45.0	51.0	−2.0	−4.0	3.0	0.41
Lipoprotein insulin resistance score	48.0	41.0	56.0	40.0	38.0	55.0	−4.0	−5.0	−1.0	0.18

*SD, standard deviation; P25, 25^th^ percentile; P75, 75^th^ percentile; VLDL, very low density lipoprotein; IDL, intermediate density lipoprotein; LDL, low density lipoprotein; HDL, high density lipoprotein; TG, triglyceride*.

* *Calculated values provided by LabCorp (Morrisville, NC, USA)*.

Changes in traditional cardiometabolic risk factors are shown in Table 4. The participants' median weight, BMI, and waist circumference decreased by 1.9 kg (*p* = 0.03), 0.7 kg/m^2^ (*p* = 0.03), and 2.0 cm (*p* < 0.01), respectively. The TC (189 to 164 mg/dl, *p* < 0.01) and LDL-c (112 to 90 mg/dl, *p* = 0.04) decreased significantly. Both HbA1c and HOMA-IR improved significantly while a decreasing trend was found in fasting insulin.

**Table 4:** Nuclear magnetic resonance spectral analysis of GlycA and lipoprotein particles and size of the nine participants.

	**Baseline**			**12 weeks**			**Changes (12 weeks - baseline)**			
	**Median**	**P25**	**P75**	**Median**	**P25**	**P75**	**Median**	**P25**	**P75**	***p*** **-value**
Weight, kg	65.3	64.0	75.3	64.0	62.1	69.2	−1.9	−5.5	0.0	0.03
BMI, kg/m2	27.9	26.0	29.4	27.0	24.9	27.9	−0.7	−2.2	0.0	0.03
Waist, cm	90.0	83.0	94.5	87.0	82.0	89.0	−2.0	−5.0	−1.0	<0.01
Hemoglobin	13.8	13.4	14.2	13.7	12.9	14.0	0.0	−0.5	0.1	0.33
Triglyceride (TG), mg/dL	139.0	96.0	186.0	138.0	103.0	166.0	−5.0	−37.0	9.0	0.36
Total cholesterol (TC), mg/dL	189.0	181.0	217.0	164.0	143.0	179.0	−22.0	−31.0	−18.0	<0.01
High-density lipoprotein (HDL-c), mg/dL	46.0	44.0	55.0	45.0	42.0	48.0	−6.0	−7.0	0.0	0.06
Low-density lipoprotein (LDL-c), mg/dL	112.0	99.0	153.0	90.0	77.0	119.0	−24.0	−27.0	−17.0	0.04
LDL/HDL ratio	1.8	1.8	3.1	2.0	1.6	2.8	−0.3	−0.3	−0.1	
Oxdized LDL, μg/dL	56.1	54.9	69.0	54.1	47.7	59.6	−2.2	−8.4	−1.5	0.13
Glucose, mg/dL	100.0	95.0	106.0	102.0	92.0	106.0	0.0	−4.0	4.0	0.98
HbA1c, %	6.0	5.8	6.3	5.7	5.6	5.9	−0.2	−0.4	−0.1	0.02
Insulin, μU/mL	11.1	10.3	14.2	7.1	5.6	12.2	−3.2	−4.8	−2.0	0.07
HOMA IR	3.2	2.2	3.7	1.8	1.4	3.2	−0.7	−0.8	−0.6	0.04
HOMA Beta	116.4	67.6	126.2	79.0	52.0	107.9	−33.3	−68.3	−5.0	0.10
hs-CRP, mg/dL	0.1	0.0	0.2	0.0	0.0	0.0	0.0	−0.1	0.0	0.25
TMAO, μM	3.1	110.3	630.5	2.4	132.8	286.5	−0.2	−262.2	67.1	0.50
Choline, μM	10.2	872.5	1217.8	7.4	645.6	896.0	−1.8	−495.0	−79.1	0.05
Carnitine, μM	25.9	3934.3	4699.4	26.0	3969.2	4761.2	0.4	−145.4	231.9	0.91
Holotranscobalamin, pmol/L	77.0	57.2	128.0	89.9	82.8	143.1	7.6	−17.4	25.5	0.57

*SD, standard deviation; P25, 25^th^ percentile; P75, 75^th^ percentile; BMI, body mass index; TG, triglyceride; HDL-C, high density lipoprotein cholesterol; LDL-C, low density lipoprotein cholesterol; HbA1c, glycated hemoglobin; HOMA-IR, homeostatic model assessment for insulin resistance; HOMA-Beta, homeostatic model assessment for beta cell function; hs-CRP, high-sensitivity C-reactive protein; TMAO, trimethylamine N-Oxide*.

**Figure 3:**
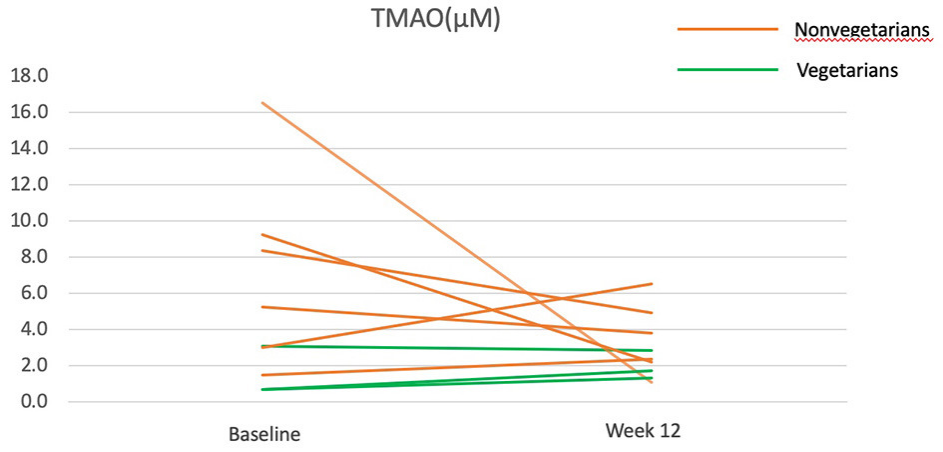
Trimethylamine N-Oxide (TMAO) before and after the 12-week diet intervention. Green lines represent participants who were vegetarians at baseline. Orange lines represent participants who were nonvegetarians at baseline.

Plasma choline, carnitine, and TMAO did not change significantly (Table 4). When examining changes at individual levels, TMAO reduction occurred mainly in participants who were previously nonvegetarians, especially those with baseline TMAO > 4μM, but stayed constant for those who were already vegetarians before the intervention (Figure 3).

**Figure 4:**
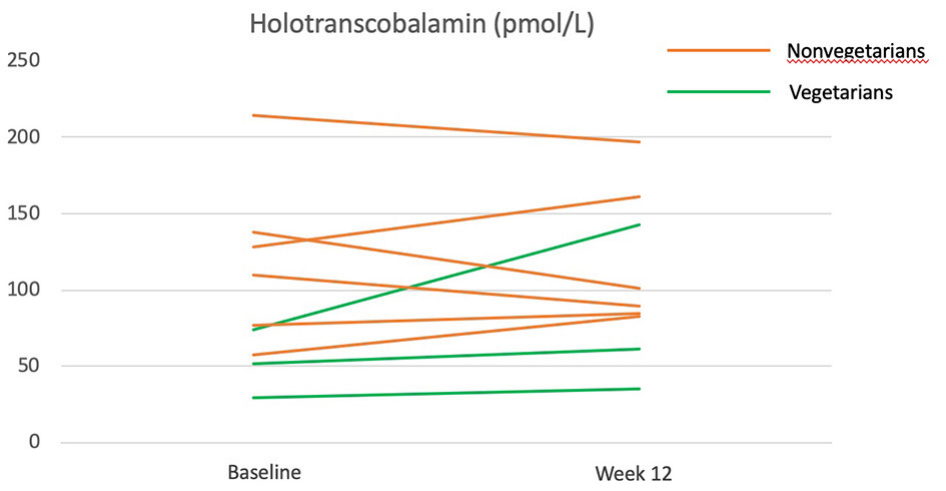
Holotranscobalamine before and after the 12-week diet intervention. Green lines represent participants who were vegetarians at baseline. Orange lines represent participants who were nonvegetarians at baseline.

Overall, the vitamin B12 status as measured by holotranscobalamin did not change significantly over the 12 weeks (Table 4). The participants who were Lacto-ovo vegetarians and had a low vitamin B12 status prior to intervention experienced an increasing trend for holotranscobalamin, while nonvegetarians were able to maintain a similar vitamin B12 status (Figure 4).

## Discussion

This 12-week dietitian-led vegan pilot intervention significantly improved GlycA, BMI, waist circumference, TC, LDL-c, LDL-p, HbA1c, and insulin resistance. A decreasing trend in TMAO was observed in those with higher baseline TMAO.

### GlycA

The significant decrease in GlycA after the intervention suggests that a whole-food vegan diet lowers systematic inflammation. This finding is consistent with previous systematic reviews and meta-analyses that assessed inflammation by CRP (10, 28). Both statin-users and non-users appeared to benefit from this healthy diet, but our study was not powered to detect heterogeneity, and larger studies are needed to confirm this finding. Several features of the dietary intervention may exert anti-inflammatory effects: high fiber and whole grains, low saturated fat, and the avoidance of ultra-processed foods.

High fiber intakes may improve gut integrity and prevent inflammation induced by the translocation of microbes and their endotoxin (29). High-fat diets may facilitate the absorption of lipopolysaccharide (an endotoxin) (29), and high saturated fat (not unsaturated fat) within the context of a high-fat diet is particularly pro-inflammatory (30). Higher fiber and lower saturated fat have been associated with higher microbiota richness, resulting in lower GlycA in pregnant women (15). A small trial supplementing inulin (a type of soluble fiber) or omega-3 fatty acid led to a decrease in GlycA (16).

Avoidance of ultra-processed foods in this dietary intervention could also have contributed to lower inflammation. Ultra-processed foods often contain emulsifiers that could disrupt gut integrity (31) and are associated with an increase in GlycA (32).

Whole-grain-rich diets (emphasized in our study) had been shown to lower body weight and systematic low-grade inflammation in a randomized crossover trial (33). Adipose tissue is an important source of chronic metabolic inflammation (29), and a recent trial and network Mendelian randomization study suggested that visceral fat mass causes an increase in GlycA *via* fasting TG (34). Although the change in TG was insignificant, fasting VLDL and chylomicron particles–major carriers of TG–decreased from 84.1 to 70.9 nmol/L (*p* = 0.05) and waist circumference (a surrogate for visceral fat) also decreased significantly (−2.0 cm, *p* < 0.01).

GlycA had been suggested to be a useful marker on the progression of rheumatic diseases such as rheumatoid arthritis, psoriasis, and systemic lupus erythematosus (13). The improvement in GlycA after a healthy vegan diet may provide a preliminary basis for hypothesis generation for these rheumatic diseases that thus far have limited nutrition therapies.

### Lipid Profile, Lipoprotein Particles, and Glucose Metabolism

Similar to previous findings of vegetarians and vegan diets (5), the 12-week program decreased TC and LDL-c substantially, with no significant change in TG, and an insignificant decrease in HDL-c. The decrease in TC in all five participants on statin therapy (data not shown), suggests that a vegan diet may further the therapeutic effect of statin.

In the NMR lipoprotein analysis, LDL-p decreased from 1,000 to 822 nmol/L (*p* = 0.02), mainly due to the change in large LDL-p (from 322 to 100 nmol/L), with no significant change in medium or small LDL-p. This may be related to avoidance of meat and reduction of saturated fat, as a feeding study also showed that meats (regardless of red or white, vs. plant protein) increased total LDL-p and large LDL-p, while lower saturated fat (vs. higher saturated fat) decreased both total LDL-p and large LDL-p (35). Although earlier studies tend to identify small LDL-p as atherogenic, evidence suggests that all LDL-p, including the large, are atherogenic (36). In the Multiethnic Study of Atherosclerosis, both large and small LDL-p is associated with carotid intima-media thickness (37).

Although the overall TG concentration did not change significantly in our study, the intervention improved insulin resistance (HOMA-IR: −0.7, *p* = 0.04), glucose (HbA1C: −0.2, *p* = 0.02), and VLDL and chylomicron particles (−23.6 nmol/L or 28%, *p* = 0.05). The effect of vegetarian and vegan diets in reducing insulin resistance is consistent in randomized controlled trials (38, 39). This improvement in insulin resistance may explain the reduction in VLDL and chylomicron particles, as insulin resistance leads to hepatic VLDL production and decreased VLDL uptake by the liver (9). Due to the small sample size, we found no differences in the subtypes of VLDL and chylomicron particles, though the largest reduction occurred in the small particles. Smaller TG-rich lipoprotein remnants may be more likely to penetrate arterial walls and contribute to atherosclerotic lesions (40). The use of monounsaturated fat (extra-virgin olive oil and pecan) may potentially contribute to a reduction in VLDL particles, as a previous trial using avocado (also a rich source of monounsaturated fat) also resulted in a similar reduction of VLDL particles (41).

Whether the small insignificant reduction in HDL-c induced by vegetarian diet should be a concern is not well studied. Our study showed that while there was a decreasing trend in HDL-c, the total HDL-p actually increased insignificantly (+0.5 μmol/L, *p* = 0.74), with a decreasing trend mainly in the small HDL-p (−1.1 μmol/L), but a slight though insignificant increase in large and medium HDL-p. As the total HDL-p and large HDL-p are inversely associated with cardiovascular diseases and are better predictors than HDL-c (42), our study suggests that a healthy vegan diet is probably unlikely to post cardiovascular risk related to HDL, particularly in the context of decreasing LDL-p. Overall, lipoprotein particle sizes did not change substantially. The slight decrease in average LDL size mainly reflects the decrease in large LDL-p. The significant *p*-value in HDL size despite no change in median may be a type 1 error.

### TMAO

The change in TMAO depends on the baseline TMAO levels. All participants with a higher baseline TMAO (> 4 μM) experienced a decrease in TMAO after the 12-week intervention but no obvious trend was found for those with lower baseline TMAO (including all three vegetarians). This finding corresponds to a previous trial, in which an increase in TMAO in response to red meat consumption appeared to be most obvious among those with baseline TMAO at the top 10%, but not those at the bottom 10% (43). As vegetarians had a low baseline TMAO, and likely have, in the long-term, nurtured non-TMAO producing gut microbiota, the shift to a vegan diet did not alter their TMAO. A previous study found that nonvegetarians are ten times more likely to be high TMAO producers than vegetarians upon carnitine challenge tests, despite no difference in fasting TMAO (17, 44) and these may be related to the presence of specific gut species (44, 45). Collectively, these results suggest that a vegan diet may be particularly beneficial for high TMAO producers.

### Bioavailability of Vitamin B12 in Taiwanese Purple Laver

A previous study had reported that Taiwanese purple laver contains true vitamin B12 rather than pseudo-vitamin B12 (23). The amount of vitamin B12 provided by Taiwanese purple laver in our study is estimated to be 3.5 μg/day from Taiwan's food composition table. In this study, vitamin B12 status was assessed by holotranscobalamin, the most sensitive and rapid-responding vitamin B12 nutritional biomarker that had previously been shown to decrease in 4 weeks of vegan diet (46). The increase of holotranscobalamin in vegetarians and the maintenance of holotranscobalamin in nonvegetarians (who have eliminated major dietary sources of vitamin B12: all animal products) during the vegan intervention suggest that the vitamin B12 in Taiwanese purple laver may be bioavailable. Studies with larger sample sizes and more comprehensive assessments (serum vitamin B12, methylmalonic acid, and homocysteine) are needed to confirm this finding.

### Limitations

The major limitation is the small sample size and the limited power to detect changes in some biomarkers, and to examine potential interaction between diet and statin on these biomarkers (given the heterogeneous status of statin use). The lack of a control group also prevents inference on causal relationships.

## Conclusion

A whole-food vegan diet instructed by registered dietitians with supplementation of healthy foods may improve both novel and traditional cardiovascular risk factors among patients with dyslipidemia. Our finding also provides preliminary evidence that Taiwanese purple laver may potentially contain bioavailable vitamin B12 for humans. All these findings need to be confirmed in larger randomized controlled trials.

## Data Availability Statement

The raw data supporting the conclusions of this article will be made available by the authors, without undue reservation.

## Ethics Statement

The studies involving human participants were reviewed and approved by Institutional Review Board at Hualien Tzu Chi Hospital. The patients/participants provided their written informed consent to participate in this study.

## Author Contributions

TC wrote the manuscript. Y-CK conducted the nutrition education session. L-YW and H-RC recruited the participants and collected the data. L-YW analyzed the data. All authors contributed to the design of this research. All authors contributed to the article and approved the submitted version.

## Funding

The study was funded by Buddhist Tzu Chi Medical Foundation (Grant Number: TCMMP106-04-03).

## Conflict of Interest

The authors declare that the research was conducted in the absence of any commercial or financial relationships that could be construed as a potential conflict of interest.

## Publisher's Note

All claims expressed in this article are solely those of the authors and do not necessarily represent those of their affiliated organizations, or those of the publisher, the editors and the reviewers. Any product that may be evaluated in this article, or claim that may be made by its manufacturer, is not guaranteed or endorsed by the publisher.
